# Season of birth and variations in male reproductive health: A population‐based cohort study

**DOI:** 10.1111/andr.70052

**Published:** 2025-04-29

**Authors:** Anne Gaml‐Sørensen, Nis Brix, Sandra Søgaard Tøttenborg, Karin Sørig Hougaard, Siri Eldevik Håberg, Mikko Myrskylä, Gunnar Toft, Jens Peter Ellekilde Bonde, Cecilia Høst Ramlau‐Hansen

**Affiliations:** ^1^ Department of Public Health Research Unit for Epidemiology Aarhus University Aarhus C Denmark; ^2^ Department of Clinical Genetics Aarhus University Hospital Aarhus N Denmark; ^3^ Department of Occupational and Environmental Medicine Bispebjerg and Frederiksberg Hospital University of Copenhagen Copenhagen NV Denmark; ^4^ Department of Public Health Section of Environmental Health University of Copenhagen København K Denmark; ^5^ National Research Centre for the Working Environment Copenhagen Ø Denmark; ^6^ Department of Public Health University of Copenhagen København K Denmark; ^7^ Centre for Fertility and Health Norwegian Institute of Public Health Oslo Norway; ^8^ Department of Global Public Health and Primary Care University of Bergen Bergen Norway; ^9^ Max Planck Institute for Demographic Research Rostock Germany; ^10^ Center for Social Data Science and Population Research Unit University of Helsinki Helsinki Finland; ^11^ Steno Diabetes Center Aarhus Aarhus University Hospital Aarhus N Denmark

**Keywords:** birth month, birth season, male fecundity, male infertility, reproductive hormones, seasonal effect, semen quality, testes volume

## Abstract

**Background:**

Season of birth has been associated with various later reproductive health outcomes in women, but little is known on the potential associations in men.

**Objectives:**

To investigate the association between season of birth and semen characteristics, testes volume and reproductive hormone levels in young men.

**Materials and methods:**

We conducted a follow‐up study of 1058 young men, born 1998 to 2000, from the Fetal Programming of Semen Quality (FEPOS) cohort, Denmark, 2017–2019. Information on season of birth was obtained from the Danish Civil Registration System, and information on male reproductive health outcomes was obtained at a clinical examination, where the men provided a semen and a blood sample and measured testes volume. Percentage differences in semen characteristics, testes volume and reproductive hormone levels were calculated according to season of birth (binary (main analysis): summer; winter and categorised by four calendar seasons and by calendar month (subanalyses)) using adjusted regression models and visualisalised according to month of birth.

**Results:**

Testosterone levels were lower (−3% (95% CI: −7%; 0%)) and oestradiol levels were higher (10% (95% CI: 2%; 20%)) in men born during the winter half‐year than the summer half‐year. The finding of higher oestradiol in men born during the winter was corroborated in analyses of calendar season and month of birth. Other reproductive health outcomes displayed some variation; however, estimates were generally close to null.

**Discussion:**

Although oestradiol levels seemed higher in men born during the winter half‐year, this could be a chance finding. Since pregnancies usually span three seasons, this finding could therefore also reflect an association between early pregnancy during the summer and oestradiol levels.

**Conclusion:**

We observed higher oestradiol levels in men born during the winter than during the summer half‐year. For the remaining reproductive health outcomes, the observed fluctuations may reflect random variation.

## 1 INTRODUCTION

Worldwide, the birth rate is declining far below the replacement rate of 2.1 births per woman.^1^, ^2^, ^3^ This decline may largely be explained by use of effective contraceptives and postponement of parenthood.^3^, ^4^ However, some studies indicate that reproductive health in both men and women may also be declining. Importantly, semen quality is currently at a very low level, at which the ability to father children is at risk.^5^, ^6^, ^7^, ^8^ Around 20% of couples in the reproductive age are affected by infertility, and up to half of these cases may be explained by male factor infertility.^9^, ^10^, ^11^, ^12^ Therefore, poor male reproductive health is a major public health concern.

The origin of poor male reproductive health remains essentially unknown^6^, ^12^; however, prenatal exposures are suggested to impair male reproductive health through fetal programming of the hypothalamic–pituitary–gonadal (HPG) axis and the reproductive organs.^6^, ^13^, ^14^, ^15^ Season of birth, which in parallel reflects season of conception, pregnancy seasonal coverage and season of the immediate postnatal environment, is associated with such exposures, including, for example, air pollution, vitamin D, light intensity, melatonin levels and infection rates that may also be associated with later poor reproductive health.^16^, ^17^, ^18^, ^19^, ^20^, ^21^

In women, season of birth has been associated with age at menarche, reproductive hormone levels, fecundity and age at menopause, and overall, women born during late spring and summer have higher oestradiol levels and seemed to be more fecund than women born during the winter.^18^ In men, one previous study found that men born during the fall had fewer children and a higher probability of being childless than men born in the spring,^21^ and another study found that early pregnancy (gestational week 8) during the winter was associated with earlier age at onset of puberty.^22^ Season of birth has yet to be examined in relation to semen characteristics, testes volume or reproductive hormone levels.

Although season of birth is difficult to apply as a direct target for prevention, the understanding of the influence of season of birth on male reproductive health may help recognize the underlying mechanisms of poor male reproductive health and point towards prenatal seasonal factors underlying poor male reproductive health. Therefore, we aimed to investigate the association between season of birth and semen characteristics, testes volume and reproductive hormone levels.

## 2 METHODS

This follow‐up study is based on the Fetal Programming of Semen Quality (FEPOS) Cohort, nested in the Danish National Birth Cohort (DNBC), an ongoing nationwide cohort of Danish women and their children with more than 25 years of follow‐up data.^23^ Sons born by women in the DNBC were eligible for invitation to FEPOS if their mother had answered the two telephone‐assisted interviews on health and health behaviours in pregnancy and had a gestational blood sample taken at their first antenatal visit at their general practitioner at their enrolment.

The men themselves had to be at least 18 years and 9 months old and live near Copenhagen or Aarhus. In total, 21,623 individuals were eligible for invitation, and 5697 young men were randomly invited to participate in FEPOS from March 2017 through December 2019. At invitation, the young men were advised to decline participation if they lacked one or both testes in the scrotum, had a vasectomy or had received chemotherapy. Of 1173 young men answering a pre‐clinical survey on health and health behaviour (corresponding to a participation rate of 21%), 1058, corresponding to a participation rate of 19% for the final study population, also attended a clinical visit (Figure S1).^24^

### 2.1 Exposure

Date of birth was obtained from the Danish Civil Registration System using the unique personal identification number that is assigned to each new‐born at birth.^25^ For practical reasons, this specific date was chosen, even though we acknowledge that season of conception, pregnancy seasons and season of the immediate postnatal environment may be important regarding reproductive health.

In the main analyses, we analysed season of birth as a binary exposure (winter half‐year: October–March, and summer half‐year: April–September). This exposure was defined according to seasonal fluctuations in environmental factors that may affect male reproductive health, such as the individuals’ vitamin D levels, melatonin, light intensity, air pollution, quality of nutrition (micronutrient content), temperature and infections.^16^, ^17^, ^18^, ^19^, ^20^, ^21^, ^26^, ^27^ In subanalyses, we analysed calendar seasons (winter: December–February; spring: March–May; summer: June–August; autumn: September–November) and calendar month (January through December).

### 2.2 Outcomes

Male reproductive health outcomes included semen characteristics, testes volume and reproductive hormone levels in blood, which was obtained at the clinical visit. The clinical visits were handled by one of the two biomedical technicians at the Department of Occupational Medicine at Aarhus University Hospital (Aarhus) or the Department of Occupational and Environmental Medicine at Bispebjerg and Frederiksberg Hospital (Copenhagen). The technicians participated in internal and external quality control schemes through the data collection, and all measurements met the standards for assessment of semen characteristics.^24^

Before their appointment at the clinic, the men were asked to be sexually abstinent for 48–72 h. Regardless of their actual abstinence time, they were included in the study. Some participants (18%) collected the semen sample at home and brought it to the clinic following specified instructions on transportation of the sample. At arrival to the clinic, or after collection of the semen sample in the clinic, the abstinence time and potential spillage of the semen sample were noted. The time of the blood sampling was also noted at the clinical visit.

#### 2.2.1 Semen characteristics

The following semen characteristics were obtained: semen volume, sperm concentration, total sperm count, motility, DNA fragmentation index (DFI), high DNA stainability (HDS) and morphology. The analyses followed the recommendations by the World Health Organisation (WHO) from 2010.^28^ Semen volume was measured by sample weight. Sperm concentration was calculated after standard sperm dilution and manual counting in duplicates. Total sperm count was calculated by multiplication of sperm concentration and semen volume. Sperm motility was calculated manually in duplicates and classified as progressive, non‐progressive or immotile spermatozoa. In the statistical models, we analysed the proportion of non‐progressive + immotile spermatozoa to ensure optimal model fit, though the primary outcome of interest was the proportion of motile spermatozoa. The proportion of spermatozoa with normal morphology was determined by the strict criteria.^24^ DFI and HDS were measured using sperm chromatin structure assay (SCSA) on thawed semen samples.^29^

#### 2.2.2 Testes volume

Following instructions, the men measured their testes volume themselves using a Prader Orchidometer, by comparing their own testes volume with 12 numbered beads of increasing size from 1 to 25 mL. This method has been found valid compared with the measurements done by an experienced examiner.^30^ The average of the two testes was used as the outcome.

#### 2.2.3 Reproductive hormones

Reproductive hormone levels were measured in serum from thawed non‐fasting venous blood samples. The following reproductive hormone levels were used as outcomes: testosterone, oestradiol, sex hormone‐binding globulin (SHBG), luteinising hormone (LH), follicle‐stimulating hormone (FSH) and free androgen index (FAI). Testosterone (limit of detection (LOD): 0.12 nmol/L, no samples below LOD) and oestradiol levels (LOD: 15 pmol/L, *n* = 87 samples below LOD) were analysed using liquid chromatography–tandem mass spectrometry (LC–MS/MS). SHBG (LOD: 0.350 nmol/L, no samples below LOD), FSH (LOD: 0.1 IU/L, *n* < 5 samples below LOD) and LH (LOD: 0.1 IU/L, *n* < 5 samples below LOD) were measured using immunoassays (Cobas 8000 e602; Roche Diagnostics, Mannheim, Germany). Samples below LOD were imputed by LOD/√2 since this is expected not to introduce systematic bias.^31^ FAI was derived by testosterone/SHBG × 100%.

### 2.3 Covariates

We used directed acyclic graphs (DAGs) to visualise the hypothesised causal framework for identification of potential confounding variables (Figure S2).^32^ From the first pregnancy interview in the DNBC, we obtained information on parental socioeconomic status assessed as highest educational level, and parental couple fecundity, including pregnancy planning, time to pregnancy (TTP) and use of medically assisted reproduction (MAR). From the Danish Medical Birth Register, we obtained information on parental age at delivery. We allowed for interaction between highest maternal and paternal educational levels, and interaction between maternal and paternal age at delivery to consider a potential differential effect of one parent's characteristics according to the other parent's characteristics. Couple fecundity was included to consider a seasonal effect on pregnancy planning: in Denmark, previous research has found that most couples pursue a pregnancy in summer compared to winter.^33^, ^34^ If highly fecund couples succeed conceiving around the time of pregnancy planning, this will result in most children being born in the spring and summer. Contrary, less fecund couple will have their conceptions delayed and will therefore more likely have children born during the fall and winter. Additionally, men of less fecund couples may have poorer reproductive health outcomes due to shared genetic or environmental components.

From the clinical visit, we obtained information on precision variables that were included in all models to enhance precision of the estimates. Semen characteristics were analysed including abstinence time (in days), place of semen sample collection (at home or at the clinic) and spillage of semen sample (yes, no). In models investigating semen volume and total sperm count, samples with spillage were not included. Interval from ejaculation to analysis (in minutes) was only included in models examining motility. Testes volume was analysed including abstinence time. Reproductive hormone levels were analysed including time of the day of blood sample drawing (morning/afternoon/evening). Categorisations of the covariates are displayed in Table 1.

**TABLE 1:** Characteristics according to season of birth in 1058 young men from the fetal programming of semen quality (FEPOS) cohort, 1998–2019, Denmark.

	Season of birth		
	Summer half‐year	Winter half‐year	
	601 (56.8)	457 (43.2)	Missings (%)
Birth month, *n* (%)			0
January	0 (0.0)	77 (16.8)	
February	0 (0.0)	73 (16.0)	
March	0 (0.0)	105 (23.0)	
April	112 (18.6)	0 (0.0)	
May	99 (16.5)	0 (0.0)	
June	102 (17.0)	0 (0.0)	
July	108 (18.0)	0 (0.0)	
August	87 (14.5)	0 (0.0)	
September	93 (15.5)	0 (0.0)	
October	0 (0.0)	96 (21.0)	
November	0 (0.0)	43 (9.4)	
December	0 (0.0)	63 (13.8)	
Maternal socioeconomic status, *n* (%)			0
High‐grade professional	98 (16.3)	62 (13.6)	
Low‐grade professional	176 (29.3)	134 (29.3)	
Skilled worker	100 (16.6)	76 (16.6)	
Unskilled worker	98 (16.3)	85 (18.6)	
Student	102 (17.0)	79 (17.3)	
Economically inactive	27 (4.5)	21 (4.6)	
Paternal socioeconomic status, *n* (%)			0
High‐grade professional	177 (29.5)	106 (23.2)	
Low‐grade professional	152 (25.3)	115 (25.2)	
Skilled worker	122 (20.3)	115 (25.2)	
Unskilled worker	82 (13.6)	72 (15.8)	
Student	34 (5.7)	26 (5.7)	
Economically inactive	34 (5.7)	23 (5.0)	
Maternal age at delivery (years), mean (SD)	31.0 (4.2)	31.0 (4.1)	∼ 0
Paternal age at delivery (years), mean (SD)	33.4 (5.6)	33.3 (5.6)	< 1
Parental couple fecundity, *n* (%)			< 1
Unplanned pregnancy	104 (17.4)	71 (15.6)	
TTP: 0 months	155 (26.0)	113 (24.8)	
TTP: 1–5 months	< 222^a^ (36.9)	< 148^a^ (32.4)	
TTP: 6–12 months	50 (8.4)	57 (12.5)	
TTP: > 12 months or MAR	70 (11.7)	68 (14.9)	
Abstinence time, *n* (%)			< 1
< 2 days	200 (33.4)	166 (36.6)	
2–3 days	< 197^a^ (32.8)	< 149^a^ (32.6)	
> 3	204 (34.1)	143 (31.5)	
Spillage, *n* (%)			< 1
No	< 490^a^ (81.5)	381 (84.3)	
Yes	111 (18.6)	71 (15.7)	
Place of semen sample collection, *n* (%)			< 1
At home	87 (14.5)	51 (11.4)	
At the clinic	< 514^a^ (85.5)	397 (88.6)	
Interval from ejaculation to analysis, *n* (%)			∼ 1
≤ 60 min	< 428^a^ (71.2)	362 (80.6)	
> 60 min	173 (29.0)	87 (19.4)	
Time at blood sample collection, *n* (%)			∼ 1
Morning < 12 p.m.	208 (35.1)	169 (37.2)	
Afternoon 12–18 p.m.	320 (54.0)	< 242^a^ (53.0)	
Evening > 18 p.m.	65 (11.0)	46 (10.1)	
Season at clinical visit			0
Winter	81 (13.5)	118 (25.8)	
Spring	104 (17.3)	101 (22.1)	
Summer	174 (29.0)	84 (18.4)	
Fall	242 (40.3)	154 (33.7)	

Abbreviations: SD, standard deviation; TTP, time to pregnancy; MAR, medically assisted reproduction.

^a^ Due to the general data protection regulation (GDPR), we have changed the numbers to avoid reporting numbers smaller than 5.

### 2.4 Statistical method

The distribution of the covariates and the male reproductive health outcomes were presented according to season of birth (summer half‐year, winter half‐year). All percentiles were presented as pseudo percentiles, which is an average of five percentiles closest to the actual percentile. This was done to comply with local regulations (General Data Protection Regulation (GDPR), Regulation (EU), 2016/679 of 25 May 2018).

Semen characteristics and testes volume were analysed according to season of birth using adjusted negative binomial regression models. This model was chosen since the outcome data were non‐normally distributed and over‐dispersed. Relative percentage differences according to exposure groups were obtained from the model as (ratios obtained from the regression models − 1) × 100% and presented with 95% confidence intervals (CIs). Reproductive hormone levels were in‐transformed and analysed according to season of birth using adjusted linear regression models. The ln‐transformation ensured optimal model fit by ensuring normally distributed residuals. Estimates and 95% CIs were back transformed and presented as percentage differences according to exposure groups. Associations with calendar month were visualised as the adjusted, average predicted values of the reproductive health outcomes in margins plots. In a sensitivity analysis, we further adjusted for season of clinical visit, since this was dependent on season of birth, due to the young men being invited when they turned 18 years and 9 months.

All models were fitted with potential confounding factors, precision variables and selection weights that were calculated as the inverse of the probability of participating in FEPOS given the potential confounding factors, maternal smoking, maternal pre‐pregnancy body mass index (BMI) and region of invitation, as described in detail previously.^35^ All models were additionally fitted with robust standard errors to account for the use of the selection weights. Model assumptions were deemed fulfilled after checking the observed distributions of the outcomes against the model‐based expected values in *Q*–*Q* plots. Data management and analyses were conducted in STATA 18.0 (StataCorp, College Station, TX, USA).

### 2.5 Ethics

The Committee for Biomedical Research Ethics in Denmark approved data collection in the DNBC ((KF) 01‐471/94). The Scientific Research Ethics Committee for Copenhagen and Frederiksberg (No. H‐16015857) and the Capitol Region of Denmark (P‐2019‐503) approved the establishment of the FEPOS cohort. This specific study was approved by the Danish Data Protection Agency (2015‐57‐0002, rec. no. 231) and the Steering Committee of the DNBC (Ref. no. 2018‐09). Written informed consent was provided by all participants at enrolment.

### 2.6 Role of the funding source

The funders of the study had no role in study design, data collection, data analysis, data interpretation or writing of the report.

## 3 RESULTS

Mean age of the men was 19 years and 3 months (standard deviation (SD): 5 months). More participants were born during the summer than the winter half‐year (Table 1). Covariates did not differ markedly across seasons of birth, however there were some indications that men born during the summer half‐year had parents with the highest socioeconomic status and the shortest TTP (Table 1). The distribution of the reproductive health outcomes is shown in Table S2.

**FIGURE 1:**
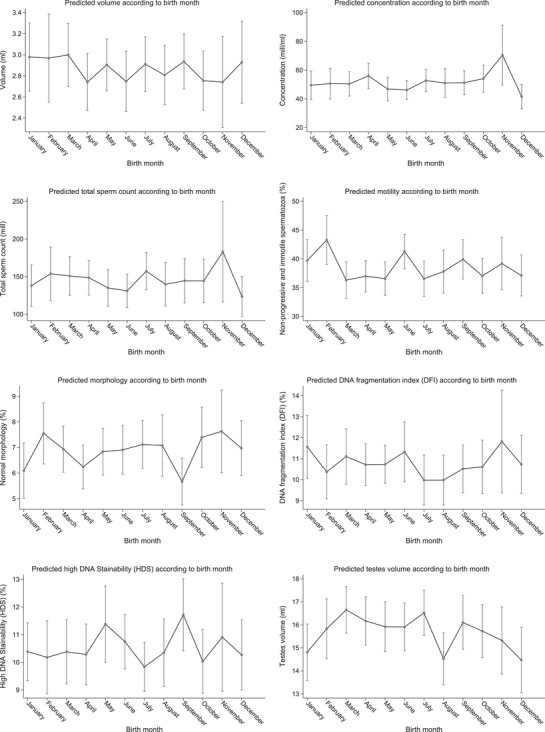
Margin plots of semen characteristics and testes volume according to month of birth in 1058 young men from the fetal programming of semen quality (FEPOS) cohort, 1998–2019, Denmark. Adjusted^a^ average predicted values of semen characteristics and testes volume based on the negative binomial regression models (grey dots) with 95% confidence intervals (grey vertical lines) for each birth month.^a^ All reproductive health outcomes are adjusted for parental socioeconomic status, parental age at delivery and parental couple fecundity. Semen characteristics were further adjusted for abstinence time, spillage and place of semen sample collection, though participants reporting spillage were excluded from models examining volume and total sperm count. The proportion of non‐progressive and immotile spermatozoa is further adjusted for interval between ejaculation and analysis. Testes volume was further adjusted for abstinence time.

**FIGURE 2:**
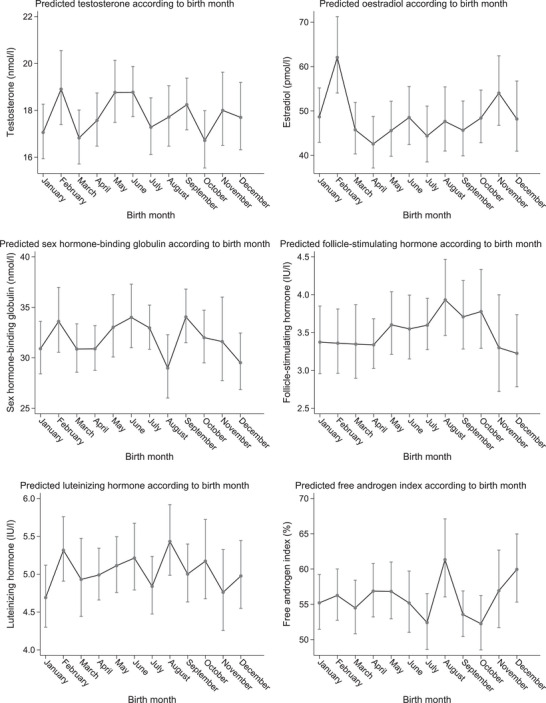
Margin plots of reproductive hormone levels according to month of birth in 1058 young men from the fetal programming of semen quality (FEPOS) cohort, 1998–2019, Denmark. Adjusted^a^ average predicted values of reproductive hormone levels based on the linear regression models (grey dots) with 95% confidence intervals (grey vertical lines) for each birth month. ^a^ Reproductive hormone levels were adjusted for parental socioeconomic status, parental age at delivery, parental couple fecundity, and time at blood sample collection.

In the main analysis, we found that testosterone was lower (−3% (95% CI: −7%; 0%)) and oestradiol was higher (10% (95% CI: 2%; 20%)) in men born during the winter half‐year than in men born during the summer half‐year. For the remaining outcomes, the estimates were close to the null (Table 2). In the subanalyses, we found that oestradiol levels were higher (13% (95% CI: 0%; 27%)) and FSH levels were lower (−10% (95% CI: ‐18%; 0%)) in men born during the winter compared with men born during the summer. For the remaining outcomes, the estimates were close to the null (Table 3). All outcomes showed variations across birth months, with no consistent patterns (Figures 1 and 2). It seemed, however, that oestradiol levels were highest and FSH levels lowest in men born during the winter (Figure 2). Results did not change when further adjusting for season of clinical visit (results not shown).

**TABLE 2:** Crude and adjusted^a^ (95% confidence intervals) relative percentage differences in reproductive health outcomes according to season of birth in 1058 young men from the fetal programming of semen quality (FEPOS) cohort, 1998–2019, Denmark.

	Season of birth		
		Winter half‐year	
	Summer half‐year	Crude	Adjusted^a^ (95% CI)
Semen characteristics^c^			
Volume (mL)^b^	Reference	−1%	2% (−4; 9)
Concentration (mill/mL)	Reference	3%	1% (−9; 13)
Total sperm count (mill)^b^	Reference	−4%	2% (−9; 15)
Motility (NP+IM%)^d^	Reference	−1%	1% (−4; 6)
Morphology (% normal)	Reference	4%	6% (−3; 16)
DFI (%)	Reference	0%	4% (−3; 12)
HDS (%)	Reference	−2%	−4% (−10; 3)
Testes volume^e^			
Average testes volume (mL)	Reference	−1%	−2% (−6; 2)
Reproductive hormones^f^			
Testosterone (nmol/L)	Reference	−4%	−3% (−7; 0)
Oestradiol (pmol/L)	Reference	5%	10% (2; 20)
SHBG (nmol/L)	Reference	−5%	−3% (−8; 2)
FSH (IU/L)	Reference	−6%	−5% (−12; 2)
LH (IU/L)	Reference	−2%	−2% (−7; 3)
Free androgen index (%)	Reference	1%	−1% (−5; 4)

Abbreviations: NP, non‐progressive; IM, immotile; DFI, DNA fragmentation index; HDS, high DNA stainability; SHBG, sex‐hormone binding globulin; FSH, follicle‐stimulating hormone; LH, luteinising hormone; IU, international units.

^a^ Adjusted for parental socioeconomic status, parental age at delivery and parental couple fecundity

^b^ Participants reporting spillage were excluded from models examining volume and total sperm count.

^c^ Further adjusted for abstinence time, spillage and place of semen sample collection. Participants reporting spillage were excluded from models examining volume and total sperm count.

^d^ Further adjusted for interval between ejaculation and analysis. Estimates represent the relative difference in the proportion of non‐progressive and immotile spermatozoa. Therefore, positive estimates should be interpreted as a relatively lower progressive motility and vice versa.

^e^ Further adjusted for abstinence time.

^f^ Further adjusted for time of blood sample drawing.

**TABLE 3:** Crude and adjusted^a^ (95% confidence intervals) relative percentage differences in biomarkers of male fecundity according to season of birth in 1058 young men from the fetal programming of semen quality (FEPOS) cohort, 1998–2019, Denmark.

	Season of birth						
		Fall		Winter		Spring	
	Summer	Crude	Adjusted (95% CI)	Crude	Adjusted (95% CI)	Crude	Adjusted (95% CI)
Semen characteristics^b^							
Volume (mL)	Reference	−7%	0% (−8; 9)	1%	5% (−4; 15)	4%	2% (−6; 10)
Concentration (mill/mL)	Reference	6%	11% (−4; 29)	−8%	−5% (−19; 10)	6%	3% (−10; 17)
Total sperm count (mill)	Reference	−5%	5% (−11; 25)	−11%	−4% (−18; 13)	7%	1% (−12; 17)
Motility (NP + IM %)^c^	Reference	−1%	0% (−7; 8)	1%	4% (−3; 12)	−5%	−5% (−11;24)
Morphology (% normal)	Reference	−5%	−4% (−16; 9)	−3%	−3% (−15; 10)	−6%	−6% (−16; 6)
DFI (%)	Reference	−2%	3% (−7; 15)	1%	5% (−6; 16)	3%	4% (−5; 14)
HDS (%)	Reference	6%	6% (−4; 16)	2%	0% (−9; 10)	5%	4% (−5; 13)
Testes volume^d^							
Average testes volume (mL)	Reference	1%	0% (−6; 7)	−3%	−4% (−10; 2)	4%	3% (−2; 9)
Reproductive hormones^e^							
Testosterone (nmol/L)	Reference	0%	−2% (−7; 4)	−1%	−1% (−7; 4)	−1%	0% (−6; 6)
Oestradiol (pmol/L)	Reference	5%	3% (−8; −16)	14%	13% (0; 27)	−3%	−5% (−15; 7)
SHBG (nmol/L)	Reference	1%	2% (−5; 10)	−4%	−2% (−9; 5)	−3%	−2% (−8; 5)
FSH (IU/L)	Reference	−3%	−1% (−11; 10)	−11%	−10% (−18; 0)	−8%	−7% (−15; 2)
LH (IU/L)	Reference	−1%	−2% (−9; 5)	−2%	−3% (−10; 4)	−1%	−3% (−9; 4)
Free androgen index (%)	Reference	−1%	−4% (−10; 2)	4%	2% (−4; 9)	2%	0% (−6; 7)

Abbreviations: NP, non‐progressive; IM, immotile; DFI, DNA fragmentation index; HDS, high DNA stainability; SHBG, sex‐hormone binding globulin; FSH, follicle‐stimulating hormone; LH, luteinising hormone; IU, international units.

^a^ Adjusted for parental socioeconomic status, parental age at delivery and parental couple fecundity.

^b^ Further adjusted for abstinence time, spillage and place of semen sample collection. Participants reporting spillage were excluded from models examining volume and total sperm count.

^c^ Further adjusted for interval between ejaculation and analysis. Estimates represent the relative difference in the proportion of non‐progressive and immotile spermatozoa. Therefore, positive estimates should be interpreted as a relatively lower progressive motility and vice versa.

^d^ Further adjusted for abstinence time.

^e^ Further adjusted for time at blood sample collection.

## 4 DISCUSSION

### 4.1 Key results

We found higher oestradiol levels in men born during the winter than in men born during the summer. For the remaining reproductive health outcomes, observed fluctuations may reflect random variation.

### 4.2 Strength and limitations

In this study, we investigated variations in male reproductive health outcomes according to season of birth in a sample of young men from the general population.^24^ Given the age of the young men, participation was likely not associated with reproductive health. Moreover, season of birth was not associated with participation. We considered selection bias due to potential selective non‐participation by applying inverse probability of selection weights in all analyses. Additionally, a validation study has examined the risk of bias due to selective non‐participation in the FEPOS cohort and found that risk of selection bias was limited.^36^

Information on season of birth was extracted from the Danish Civil Registration System and is therefore considered valid. Reproductive health outcomes were measured using state‐off‐the‐art laboratory technics that were continuously quality controlled. However, non‐differential measurement errors and the with‐in individual variation in semen characteristics may cause bias towards the null.^37^ Any measurement errors in self‐assessment of testes volume are likely also non‐differential regarding season of birth, and may thus cause bias towards the null. Although we adjusted for time of the day of blood sampling to accommodate the daily fluctuations in reproductive hormone levels, day‐to‐day variations may also cause bias towards the null.

We adjusted for maternal and paternal socioeconomic status and age at delivery in addition to parental couple fecundity to obtain exchangeability between men born in different seasons due to differences in pregnancy planning and parental fecundity. Although we still risk residual confounding, differences in pregnancy planning have been found to be small, for example, Basso et al. found that pregnancy planning peaked during the summer (30%) compared to the winter (21%).^34^

### 4.3 Interpretation

The HPG axis and the reproductive organs may be particularly vulnerable towards interferences in fetal life.^6^, ^15^ Here, the gonadotropin‐releasing hormone (GnRH) releasing neurons and the gonadotropin producing cells develop in the pituitary gland, and human chorionic gonadotropin stimulates the fetal Leydig cells to initiate testosterone secretion.^38^, ^39^, ^40^ Following this, GnRH released from the hypothalamus stimulates LH release from the pituitary gland that stimulates the production of testosterone further.^38^, ^39^ Simultaneously, the release of FSH from the pituitary gland stimulates the Sertoli cells in the fetal testes to proliferate.^38^, ^39^, ^41^ In males, gonadotropin levels peak in mid gestation, after which the activity decreases due to suppression by placental oestrogens.^38^, ^39^ The HPG axis is suppressed at term, but is reactivated after birth, causing the testes to produce testosterone.^38^, ^39^ After approximately 6 months, the HPG axis activity decreases and enters a quiescent state until puberty.^38^ Therefore, the male reproductive system may be vulnerable throughout prenatal life and the immediate post‐birth period, and associations between season of birth may reflect association between season of early pregnancy, later pregnancy or even season after the birth. This complicates the interpretation of our findings. It appeared, however, that birth during the winter half‐year was associated with higher oestradiol than birth during the summer half‐year. High oestradiol levels may inhibit sperm cell production,^42^ indicating a potential disadvantage in men born during the winter regarding reproductive health and fecundity. This finding aligns with research in women: women born during the summer had higher oestradiol levels and seemed to be more fecund than women born during the winter.^18^ This too aligns with the hypothesis that men of less fecund couples are born during the winter to a higher extent and may have poorer reproductive health outcomes due to shared genetic or environmental components. However, further evidence is required to confirm this finding to rule out the possibility that the pattern occurred by chance.

Additionally, the external environment changes through the seasons, further complicating the interpretation of the observed season of birth variations. Many environmental factors fluctuate during seasons, and the results can therefore not be ascribed specific factors.^18^, ^19^, ^20^, ^26^, ^27^ The winter half‐year is characterised by, for example, low vitamin D levels, melatonin, and high risk of infections compared to the summer half‐year, why the winter half‐year was regarded as the exposure and therefore analysed relative to the summer half‐year.

Besides being associated with gestational conditions in the mother,^43^ season of birth has also been associated with multiple different outcomes in the offspring later in life, including biological effects (e.g., birth weight, cardiometabolic disorders, timing of puberty), and cultural effects (e.g., sports performance, educational attainment) that may affect reproductive health directly or through life choices.^21^, ^22^, ^26^, ^43^, ^44^, ^45^, ^46^ We did not investigate potential mediating paths in this study.

In conclusion, we found variations in male reproductive health outcomes according to season of birth in this cohort of young men from the general population of Denmark. Particularly, we saw indications of higher oestradiol levels in men born during the winter relative to men born during the summer. Unfortunately, we were not able to conclude, whether this was observed due to genetic or environmental components shared in families, or whether the observed associations followed seasonal fluctuations in the external environment during potential sensitive periods in fetal life or following birth. Since the seasonal changes in the external environment differ according to geographic area and latitude, our results are primarily generalizable to other populations from the Western world at a similar latitude.

## AUTHOR CONTRIBUTIONS

The funding for FEPOS was acquired by Sandra Søgaard Tøttenborg and Jens Peter Ellekilde Bonde. Cecilia Høst Ramlau‐Hansen acquired the funding for this specific study. The data collection in FEPOS was planned and headed by Gunnar Toft, Sandra Søgaard Tøttenborg, Karin Sørig Hougaard, Jens Peter Ellekilde Bonde and Cecilia Høst Ramlau‐Hansen. Anne Gaml‐Sørensen planned this study and designed the analytic strategy with Cecilia Høst Ramlau‐Hansen. Anne Gaml‐Sørensen performed the statistical analyses and wrote the first draft. All authors interpreted the data, revised the manuscript critically, approved and accepted responsibility of the final manuscript.

## CONFLICT OF INTEREST STATEMENT

The authors have no conflicts of interest.

## Supporting information



Supporting information

## Data Availability

The dataset analysed in the study is not publicly available due to national data security legislation on sensitive personal data. Researchers may apply for access to data from the DNBC. Please see https://www.dnbc.dk/data‐available or write to dnbc-research@ssi.dk for additionally information.
