# Supplementary material for: Season of birth and variations in male reproductive health: A population‐based cohort study
Source: Andrology. 2025 Apr 29;14(1):36–47. doi: 10.1111/andr.70052 (PMC12670485; doi:10.1111/andr.70052)
Supplement: Supplementary file 1 — Supporting information [file ANDR-14-36-s001.docx]

**Supplementary Figure S1**

Flowchart of the inclusion of study participants in the study investigating season of birth variations in male reproductive health, the Fetal Programming of Semen Quality (FEPOS) Cohort, Denmark, 1996- 2019.

| n = 5697 young men invited to the FEPOS Cohort | | |  |  |  |  |
| --- | --- | --- | --- | --- | --- | --- |
|  |  |  |  | Young men not responding to the FEPOS pre-clinical survey (n = 4524) | | |
|  |  |  |  |  |  |  |
| n = 1173 young men answered the FEPOS pre-clinical survey | | |  |  |  |  |
|  |  |  |  | Young men not attending the clinical visit (n = 115) | | |
|  |  |  |  |  |  |  |
| n = 1058 young men participated in FEPOS | | |  |  |  |  |

**Supplementary Figure S2**

Directed acyclic graph illustrating the assumed causal framework underlying the study investigating season of birth variations in male reproductive health.

Boxes indicate adjustment in the statistical models.
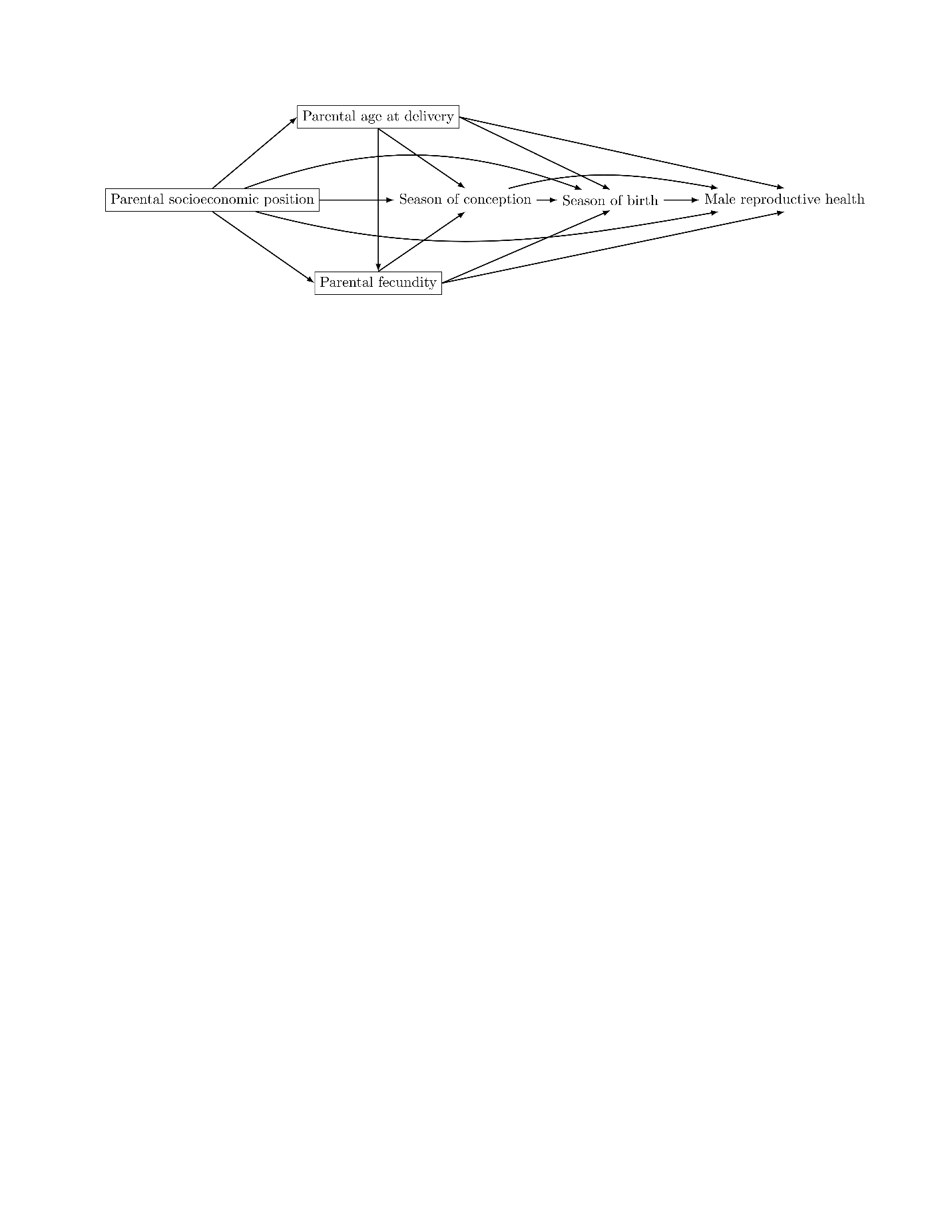


| **Supplemental Table S1.** Reproductive health outcomes^a^ according to season of birth in 1058 young men from the Fetal programming of Semen Quality (FEPOS) Cohort, 1998-2019, Denmark | | |  |
| --- | --- | --- | --- |
|  |  |  |  |
|  | Season of birth | |  |
|  | Summer half-year | Winter half-year |  |
| n (%) | 601 (56.8) | 457 (43.2) |  |
| Semen characteristics |  |  |  |
| Volume (ml) | 2.7 (1.9; 3.7) | 2.6 (1.9; 3.6) |  |
| Concentration (mill/ml) | 39.8 (19.7; 70.0) | 37.1 (18.3; 74.3) |  |
| Total sperm count (mill) | 103.6 (47.4; 202.6) | 101.2 (39.9; 195.5) |  |
| Motility (PR %) | 63.0 (52.0; 73.0) | 63.7 (53.0; 74.0) |  |
| Morphology (% normal) | 6.0 (3.0; 10.0) | 6.0 (4.0; 10.0) |  |
| DFI (%) | 9.8 (7.0; 13.0) | 9.3 (7.0; 13.7) |  |
| HDS (%) | 9.0 (7.0; 13.0) | 9.0 (6.0; 13.0) |  |
| Testes volume |  |  |  |
| Average testes volume (ml) | 15.0 (12.0; 20.0) | 15.0 (12.0; 20.0) |  |
| Reproductive hormones |  |  |  |
| Testosterone (nmol/L) | 18.5 (15.1; 22.0) | 17.6 (14.5; 22.0) |  |
| Oestradiol (pmol/l) | 51.4 (33.1; 72.8) | 53.6 (36.5; 73.9) |  |
| SHBG (nmol/l) | 33.0 (26.0; 42.0) | 32.0 (24.0; 40.0) |  |
| FSH (IU/l) | 3.6 (2.5; 5.2) | 3.5 (2.4; 4.7) |  |
| LH (IU/l) | 5.1 (4.0; 6.6) | 5.1 (3.9; 6.5) |  |
| Free androgen index (%) | 55.5 (44.7; 68.7) | 55.6 (46.3; 68.9) |  |
| Abbreviations: p50, 50th pseudo percentile. IQR, pseudo intra quartile range. DFI, DNA fragmentation index. HDS, high DNA stainability. SHBG, Sex-hormone binding globulin. FSH, Follicle-stimulating hormone. LH, Luteinizing hormone. IU, international units. | | |  |
|  |  |  |  |
| ^a^ The biomarkers of male fecundity are presented as p50 (IQR). All percentiles are pseudo percentiles calculated from the average of the five values nearest to the actual percentile | | |  |
